# Inversion of the imprinting control region of the *Peg3* domain

**DOI:** 10.1371/journal.pone.0181591

**Published:** 2017-07-18

**Authors:** Joomyeong Kim, Hongzhi He, Hana Kim

**Affiliations:** Department of Biological Sciences, Louisiana State University, Baton Rouge, LA, United States of America; Peking University Third Hospital, CHINA

## Abstract

The imprinting of the mouse *Peg3* domain is controlled through a 4-kb genomic region encompassing the bidirectional promoter and 1^st^ exons of *Peg3* and *Usp29*. In the current study, this ICR was inverted to test its orientation dependency for the transcriptional and imprinting control of the *Peg3* domain. The inversion resulted in the exchange of promoters and 1^st^ exons between *Peg3* and *Usp29*. Paternal transmission of this inversion caused 10-fold down-regulation of *Peg3* and 2-fold up-regulation of *Usp29* in neonatal heads, consistent with its original promoter strength in each direction. The paternal transmission also resulted in reduced body size among the animals, which was likely contributed by the dramatic down-regulation of *Peg3*. Transmission through either allele caused no changes in the DNA methylation and imprinting status of the *Peg3* domain except that *Zfp264* became bi-allelic through the maternal transmission. Overall, the current study suggests that the orientation of the Peg3-ICR may play no role in its allele-specific DNA methylation, but very critical for the transcriptional regulation of the entire imprinted domain.

## Introduction

A subset of mammalian genes are functionally different between two alleles due to genomic imprinting, by which one allele is repressed through epigenetic mechanisms [1, 2]. About 100 to 200 genes are known to be imprinted so far, and these genes tend to play significant roles in the biological pathways controlling fetal growth rates and maternal-caring behaviors [1, 2]. Also, genomic imprinting is mainly found within eutherian mammals, thus believed to have co-evolved with their unusual reproductive scheme involving placentation and viviparity [3–6]. Imprinted genes are clustered in specific regions of chromosomes, forming imprinted domains. In a given domain, a small genomic region, termed ICR (Imprinting Control Region), is responsible for controlling the transcription and allele-specific expression of individual genes [1, 2]. There are two types of ICRs based on their genomic positions relative to the associated genes [2, 7, 8]. Some ICRs are localized in intergenic regions, including the ICRs of *H19*/*Igf2*, *Gtl2*/*Dlk1* and *Rasgrf1* domains. Interestingly, these ICRs are all methylated during spermatogenesis. On the other hand, all the ICRs that are methylated during oogenesis tend to be localized very closed to or part of the promoter regions of the associated imprinted genes [2, 7, 8]. The functional implication for this bias between the two types of ICRs is currently unknown.

*Peg3* (paternally expressed gene 3) is a founding member of the mammalian *Peg3* imprinted domain, which covers an evolutionarily well-conserved 500-kb genomic region in human chromosome 19q13.4/ proximal mouse chromosome 7 [9–11]. This domain contains paternally expressed *Peg3*, *Usp29*, *APeg3*, *Zfp264* and maternally expressed *Zim1*, *Zim2*, *Zim3* [12]. The majority of these genes except *Zim3* are known to be expressed in neonatal heads [12]. The *Peg3* domain is also controlled through an ICR, termed the Peg3-DMR (Differentially Methylated Region) [13–15]. The Peg3-DMR obtains DNA methylations as a gametic signal during oogenesis, and maintains its allele-specific methylation pattern throughout the lifetime of mammals. This ICR covers a 4-kb genomic region that harbors a bidirectional promoter for *Peg3* and *Usp29* and also the 1^st^ intron region of *Peg3* with a tandem array of 7 YY1 binding sites [16]. According to recent results, deletion of this ICR resulted in global changes in the transcriptional levels and mono-allelic expression patterns of the entire domain, confirming that this region is indeed a major controlling region for the *Peg3* domain [15]. Also, YY1 binding sites have been shown to control the transcriptional levels of *Peg3* and the other imprinted genes [17–19]. It is, however, still unknown the detailed mechanisms by which this 4-kb ICR controls the transcription and imprinting of the individual genes that are distributed throughout the 500-kb *Peg3* domain.

As part of ongoing efforts, the ICR of the *Peg3* domain was further characterized in the current study. Since this 4-kb region contains the bidirectional promoter for *Peg3*/*Usp29* and also an unusual tandem array of YY1 binding sites, we decided to test the orientation dependency of this ICR for its regulatory roles in the transcription and imprinting of the entire domain. According to the results, the inversion of this ICR caused global changes in the transcriptional levels of several genes, including *Peg3*, *Usp29* and *Zfp264*. In contrast, the inversion had no impact on the DNA methylation and imprinting status of the individual genes. More detailed results have been presented below.

## Results

### Generation of the inverted allele of the Peg3-DMR

**Fig 1:**
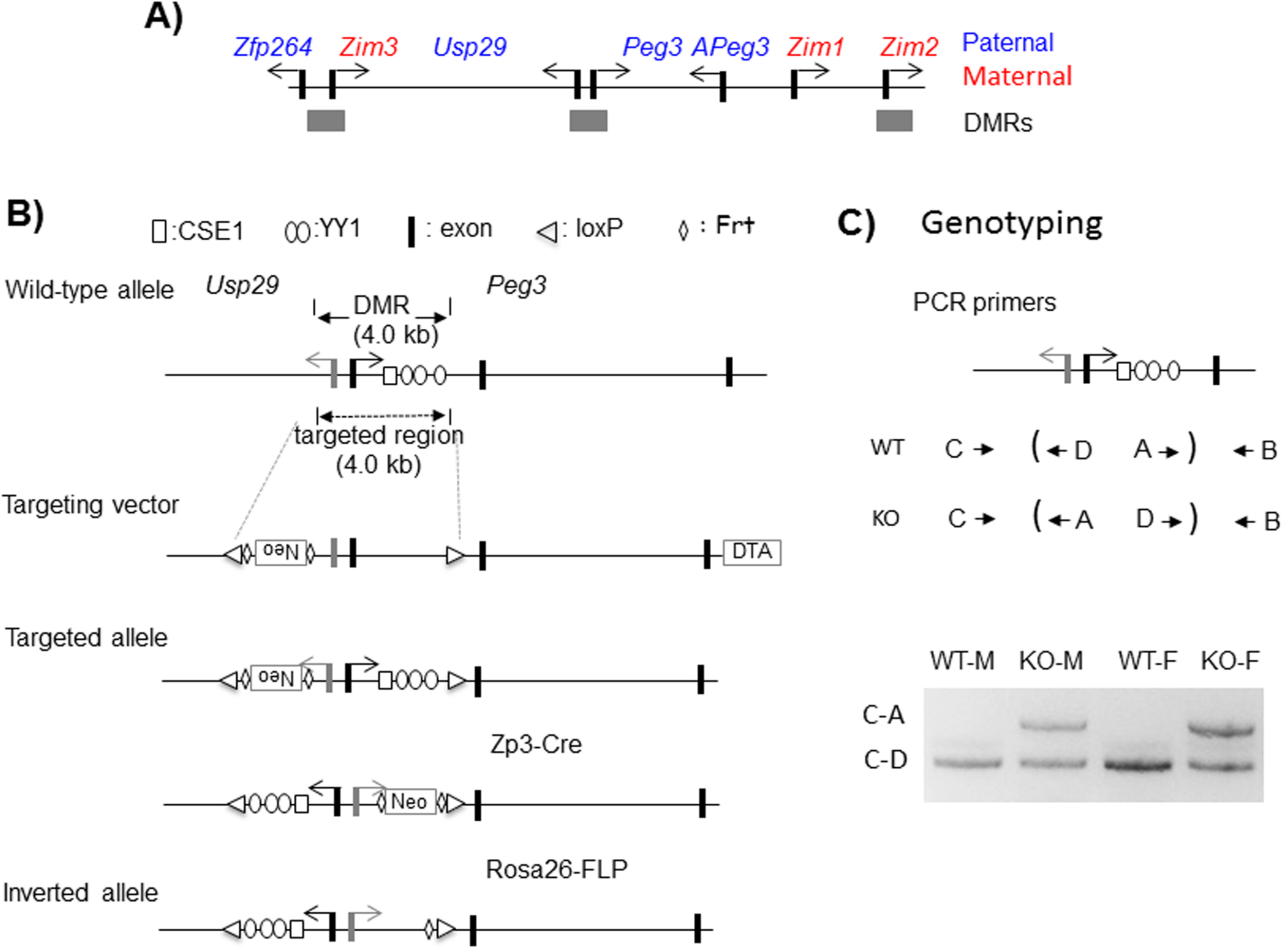
*Peg3* domain and inversion scheme. (**A**) Schematic representations of the *Peg3* domain. Each imprinted gene is indicated with an arrow. The paternally and maternally expressed genes are indicated with blue and red, respectively. The three DMRs are indicated with gray boxes. (**B**) The 4.0-kb Peg3-DMR contains the first exons of *Peg3* and *Usp29* and the 2.5-kb 1^st^ intron region of *Peg3* with multiple YY1 binding sites. The transcriptional direction of *Peg3* and *Usp29* is indicated with arrows, and exons are indicated with thick vertical lines. The two loxP sites flanking the 4-kb Peg3-DMR are indicated with triangles. The region corresponding to the neomycin resistance gene (*NeoR*) along with the two flanking FRT sites are indicated by an open box and diamonds, respectively. (**C**) PCR-based genotyping. The upper panel indicates the positions and orientations of the 4 primers used for genotyping of the inverted allele. Each set of primers is designed to target one loxP site: the loxP site in the *Usp29* direction by C and D whereas the one in the *Peg3* direction by A and B. The bottom panel shows a set of genotyping results demonstrating the successful inversion of the Peg3-DMR. A set of 4 individual DNA were amplified with three primers, C, D and A. The product by C and D represents the amplified product from the wild-type allele, whereas the product by C and A represents the amplified product from the inverted allele.

The Peg3-DMR covers a 4-kb genomic region harboring the 1.5-kb bidirectional promoter for *Peg3* and *Usp29* and also the 2.5-kb 1^st^ intron region of *Peg3* (**Fig 1A and 1B**). This region has been previously targeted through conditional KO experiments utilizing the Cre-loxP recombination system [15, 20]. Some of these previous experiments were also performed with the KO construct containing two loxP sites with opposite orientation for potential inversion experiments (**Fig 1B)** [15, 20]. The targeted ES clones have been used for establishing one mutant mouse line, termed Peg3^KO1^, which has been further bred with two different recombinase lines. The mutant line was first crossed with the Zp3-cre line, resulting in the inversion of the 4-kb Peg3-DMR through the two loxP sites. This mutant line with the inversion was subsequently bred with the Rosa26-FLP line to remove the *NeoR* (Neomycin Resistance) cassette through FLT sites. The proper inversion and integrity of the mutant allele was further confirmed through PCR-based genotyping using the genomic DNA isolated from the mutant animals (**Fig 1C**). In sum, we have successfully established a mutant line with the inverted allele of the Peg3-DMR through a series of breeding experiments involving two recombination systems.

### Formation of two fusion transcripts

**Fig 2:**
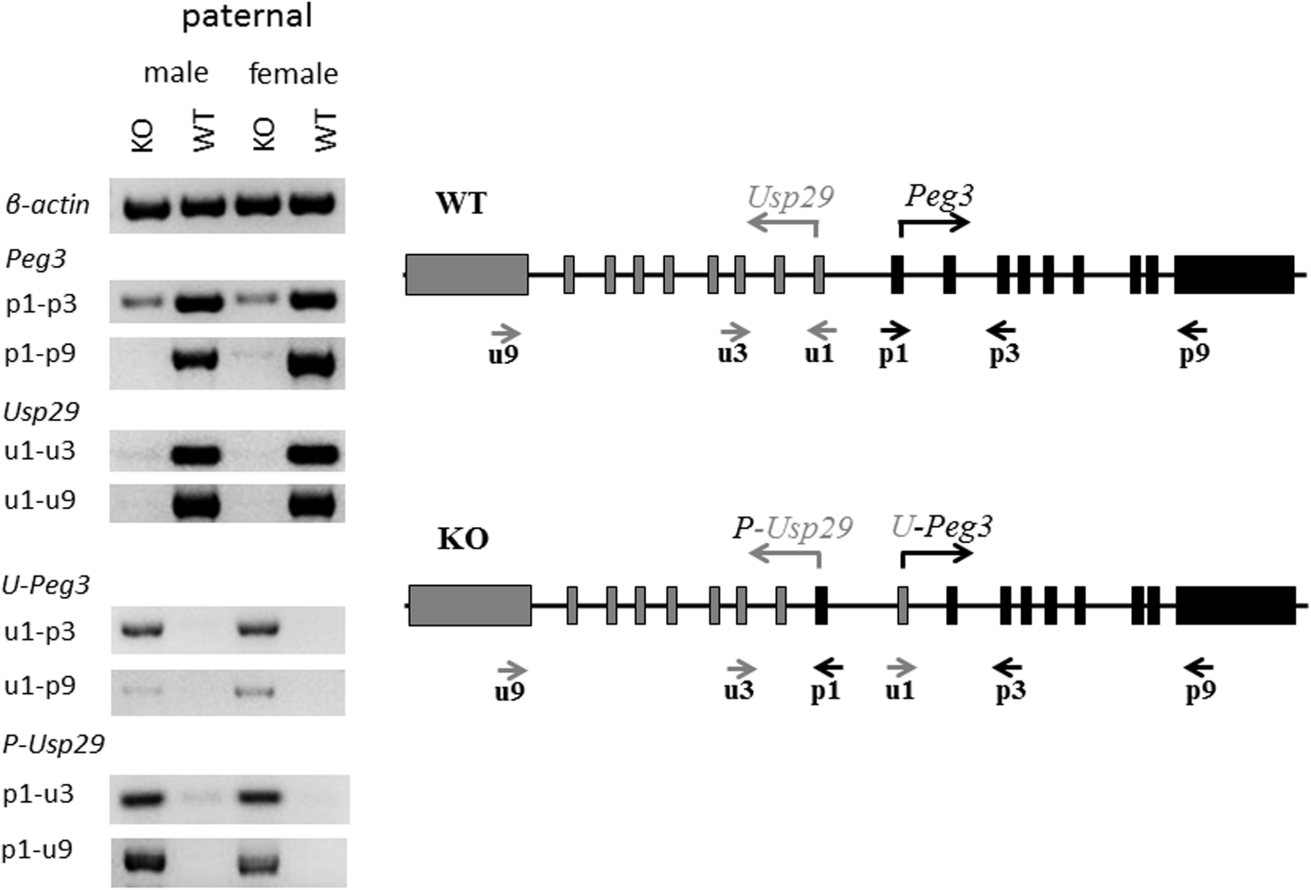
Identification of two fusion transcripts. Total RNA isolated from the heads of one-day-old heterozygotes with the paternal transmission of the inverted allele were used for detecting two fusion transcripts. (right panel) Two sets of primers were used for this RT-PCR-based experiments: the first set of three primers (p1,p3,p9) target the exons of *Peg3* whereas the second set of three primers (u1,u3,u9) target the exons of *Usp29*. The relative orientations and positions of these primers are indicated with arrows underneath the exon structures of *Peg3* and *Usp29*. (left panel) A set of cDNA prepared from the KO and WT of both sexes with the paternal transmission of the inverted allele were tested with various primer sets: including an internal control (β-actin), *Peg3* sets (p1-p3, p1-p9), *Usp29* sets (u1-u3, u1-u9), *U-Peg3* sets (u1-p3, u1-p9) and *P-Usp29* sets (p1-u3, p1-u9).

As an initial step, we tested whether the inverted bidirectional promoter is still functional for deriving the transcription of both *Peg3* and *Usp29* (**Fig 2**). For this series of expression analyses, total RNA was isolated from the head portion of one-day-old neonates inheriting the inverted allele paternally, which had been generated from the crossing of male heterozygotes with female littermates. This set of total RNA isolated from the KO and WT of both sexes were converted to a set of cDNA, which were then used for a series of RT-PCR-based surveys detecting potential transcripts from the inverted allele. On a separate note, the imprinted genes in the *Peg3* domain are sexually biased in terms of their expression levels [21], thus this series of analyses included the mice with both sexes. This survey used two sets of primers targeting the exons of *Peg3* and *Usp29*: the primers targeting *Peg3* exons include p1, p3, p9, whereas those targeting *Usp29* exons include u1, u3, u9. The relative orientations and positions of these primers are indicated in **Fig 2**. The results from this series of surveys are summarized as follows. First, the inverted bidirectional promoter was functional for generating the transcripts for both *Peg3* and *Usp29*. In the *Peg3* direction, the inverted promoter generated a fusion transcript that was comprised of the 1^st^ exon of *Usp29* and the 2^nd^ through 9^th^ exons of *Peg3*, termed *U-Peg3*. In the *Usp29* direction, the inverted promoter also produced another transcript that was made of the 1^st^ exon of *Peg3* and the 2^nd^ through 9^th^ exons of *Usp29*, termed *P-Usp29*. The proper exon joining and combinations for both fusion transcripts were independently confirmed through sequencing the RT-PCR products of *U-Peg3* and *P-Usp29*. It is interesting to note that very low levels of *Peg3* expression were also detected from the KO samples (p1-p3 of KO in **Fig 2**), suggesting low levels of de-repression of the maternal allele of the Peg3-DMR. Second, the expression levels of these fusion transcripts turned out to be different from the levels of their original transcripts. In particular, the expression levels of *U-Peg3* transcript detected from KO were much lower than those detected from the original transcript of *Peg3* in WT (u1-p3 or u1-p9 of KO versus p1-p3 or p1-p9 of WT in **Fig 2**). In contrast, the expression levels of the *P-Usp29* transcript in KO were comparable to those detected from the original *Usp29* transcript in WT (p1-u3 or p1-u9 in KO versus u1-u3 or u1-u9 in WT). It has been known that the spatial expression patterns of *Peg3* and *Usp29* are very similar, but that the levels of *Peg3* are much greater than those from *Usp29* [20, 22]. Thus, the observed difference from the inverted promoter might be reflecting the fact that the bidirectional promoter may have different promoter strengths between the two different directions with the *Peg3* direction showing much greater strength than the *Usp29* direction. Third, the inverted promoter was shown to be non-functional when inherited as a maternal allele (**S1 File**). We performed a similar series of analyses using the total RNA isolated from the mutants with the maternal transmission of the inverted allele. Despite numerous attempts, however, we did not detect any levels of the fusion transcripts, suggesting that the maternal allele with the inversion may be still subject to transcriptional repression as part of genomic imprinting [12]. We also tested the presence of potential fusion transcripts between the bidirectional promoter and the adjacent genes, including *Zim1*, *Zim2* and *Zfp264*, but this series of analyses did not derive any fruitful outcome, no fusion transcript so far (**S2 File**). Taken together, this series of surveys confirmed that the inverted bidirectional promoter is functional and produces the two fusion transcripts, *U-Peg3* and *P-Usp29*, with different expression levels.

### Effects on the expression levels of the imprinted genes

**Fig 3:**
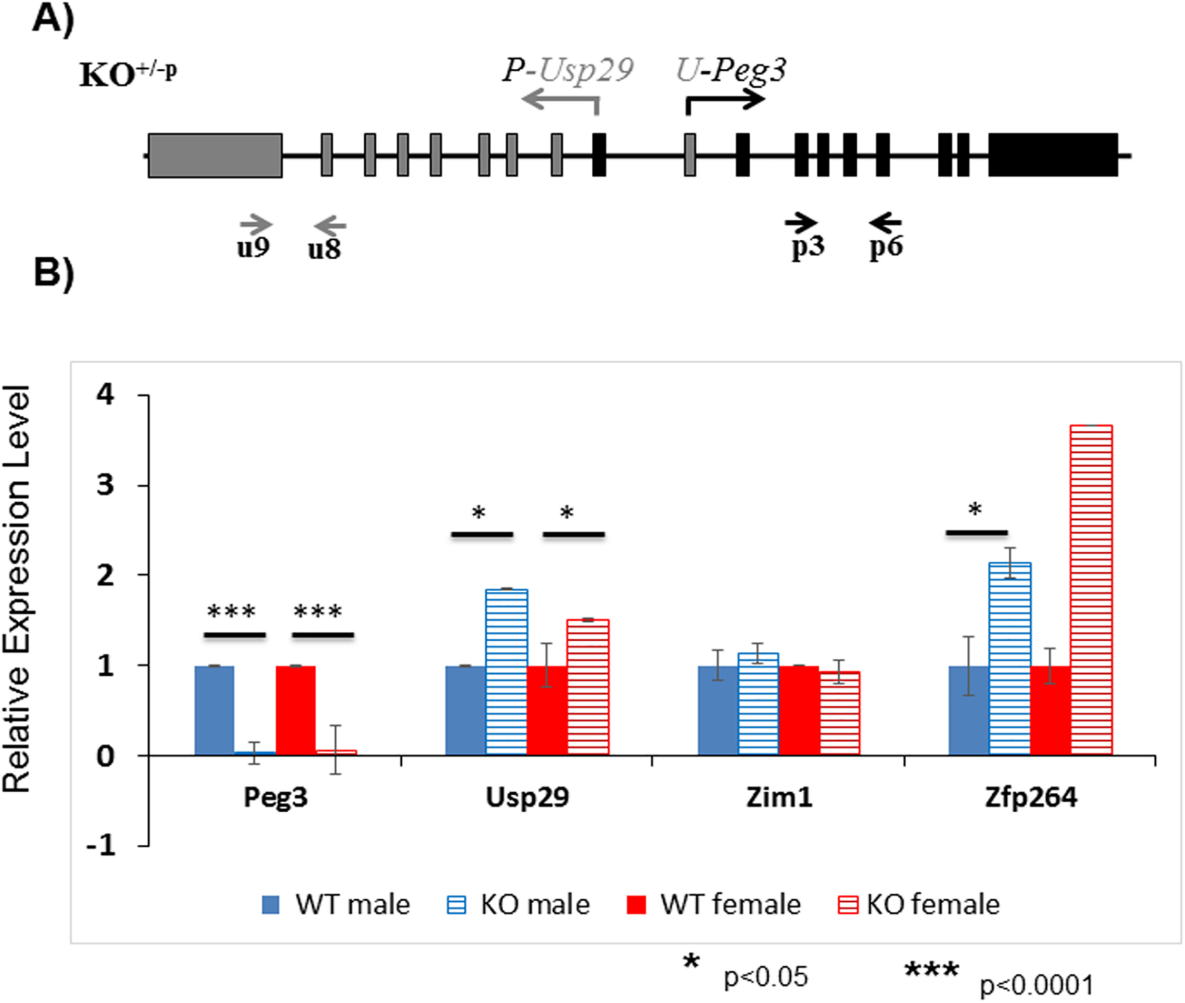
Effects of the inversion on the expression levels of the *Peg3* domain. Potential effects of the inversion on the expression levels of the individual genes within the *Peg3* domain were analyzed using a set of total RNA isolated from the heterozygotes of both sexes with the paternal transmission of the inverted allele. (**A**) This series of qRT-PCR analyses used several primer sets targeting the four imprinted genes, including *Peg3*, *Usp29*, *Zim1* and *Zfp264*. In the case of the *Peg3* and *Usp29*, the expression levels of both the fusion and original transcripts were simultaneously measured through two sets of primers targeting the shared exons between the fusion and original transcripts. (**B**) The expression levels of each gene were first normalized with an internal control (β-actin), and the subsequent value was compared between KO versus WT. The relative levels are presented in a graph with standard errors (S.E.). This set of analyses were repeated using three biological replicates.

The expression levels of the fusion transcripts driven by the inverted promoter were further analyzed using a series of qRT-PCR analyses (**Fig 3**). This series of expression analyses employed an almost identical strategy as described above except the fact that the expression levels of *Peg3* and *Usp29* were measured through the different primer sets targeting the shared exons between the fusion and original transcripts, thus allowing unbiased comparison of the expression levels of the two types of the transcripts (**Fig 3A**). The results from this series of analyses are summarized as follows. First, the expression levels of both *Peg3* and *Usp29* detected in the KO samples were quite different from those of the original transcripts in the WT samples. The expression levels of *U-Peg3* were 10-fold lower than the levels of *Peg3* in both males and females. In contrast, the expression levels of *P-Usp29* were 2.0- and 1.5-fold greater than those of the *Usp29* in females and males, respectively (**Fig 3B**). Thus, the inversion appeared to have caused two different outcomes for the two genes, down-regulation for *Peg3* and up-regulation for *Usp29*. This is also consistent with the patterns seen in the initial set of surveys (**Fig 2**). Second, the expression levels of the neighboring genes were also affected by the inversion of the Peg3-DMR. The expression levels of *Zfp264* in KO showed 2 and 3.5-fold greater than those from WT, indicating up-regulation for *Zfp264*. In contrast, the expression levels of *Zim1* were not affected at all in both sexes, showing almost similar levels between the KO and WT samples. Third, we also performed a similar series of expression analyses using a set of total RNA isolated from the animals with the maternal transmission of the inverted allele, which did not show any major changes in their expression levels (**S3 File**). We have repeated this series of experiments using three independent sets of biological replicates, which derived a consistent conclusion as described above. This series of expression analyses did not include two genes, *Zim2* and *Zim3*, mainly due to their very low expression levels in the neonatal heads. In sum, this series of expression analyses concluded that the inverted bidirectional promoter resulted in significant changes in the expression levels of the three genes, down-regulation of *Peg3* and up-regulation of *Usp29* and *Zfp264*.

### Effects on the DNA methylation and imprinting status of the *Peg3* domain

**Fig 4:**
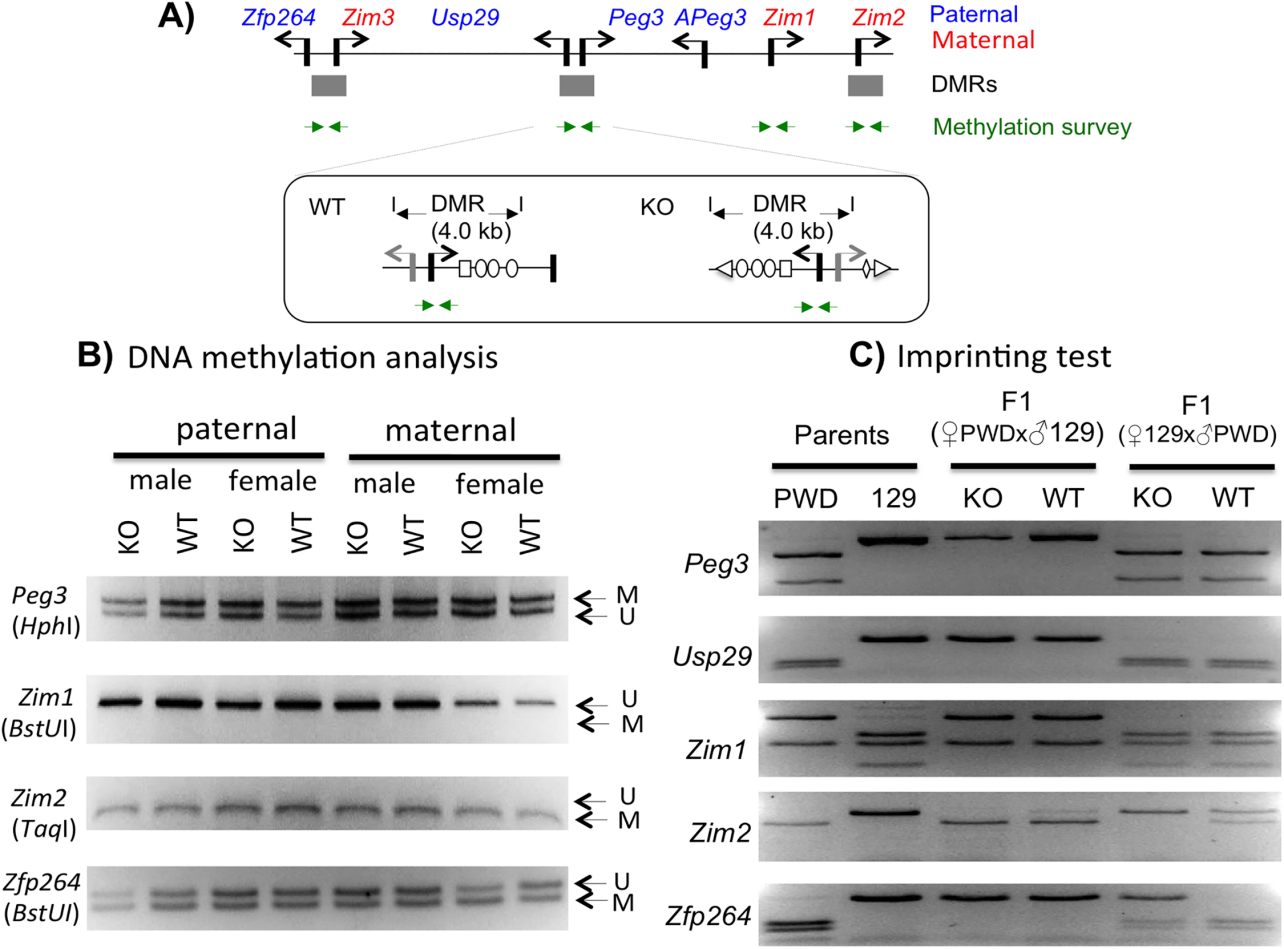
Effects on the DNA methylation and imprinting status of the *Peg3* domain. (**A**) Schematic representation of the *Peg3* domain showing the relative positions of 4 genomic intervals that have been analyzed for their DNA methylation status. The relative position of the region targeting the Peg3-DMR is illustrated in detail within a box. The 400-bp region surrounding the 1st exon of *Peg3* was analyzed, which is localized within the inverted region of the KO allele. (**B**) A series of DNA methylation analyses were performed using the DNA isolated from the neonatal heads. The isolated DNA was treated with the bisulfite conversion protocol, and subsequently used for the amplification of each target region. The amplified PCR product was analyzed with COBRA. The restriction enzyme used for each set of PCR products is shown underneath of the name of each target. The unmethylation and methylation based on the digestion pattern by a given restriction enzyme are also indicated by the letter U and M with arrows, respectively. (**C**) RT-PCR-based imprinting test of the genes within the *Peg3* domain. This series of imprinting tests used the total RNA isolated from the neonatal heads of the F1 hybrid of the male set that had been prepared through the reciprocal crossing of the heterozygotes with the 129/B6 background and the breeding partners with the PWD/PhJ background. The products from RT-PCR were digested with a given restriction enzyme to differentiate parental alleles, which are shown as different-size DNA fragments on gel images. The two columns on the left represent the digestion patterns for two parental strains for each gene; the two middle columns represent the results from the F1 hydrid set with the paternal transmission of the KO allele (male heterozygote with female PWD/PhJ); the two columns on the right represent the results from the F1 hybrid set with the maternal transmission of the KO allele (male PWD/PhJ with female heterozygote).

We also tested whether the inversion of the Peg3-DMR has any impact on the DNA methylation status of the *Peg3* domain using the following two strategies. First, we performed DNA methylation analyses using the DNA isolated from the head portion of the neonates with the paternal and maternal transmission of the inverted allele. A set of DNA panel representing the KO and WT of both sexes with both transmissions were treated with the bisulfite conversion protocol [23], and the converted DNA were used for targeting the three DMRs along with the promoter region of *Zim1* (**Fig 4A**). The amplified products were digested with several restriction enzymes that can differentiate the DNA methylation status of the original DNA [24]. According to the results, the inversion did not cause any changes in the DNA methylation status of three DMRs, showing no difference between the KO and WT samples with both paternal and maternal transmissions (**Fig 4B**). This suggests that the inversion may have no impact on the establishment and maintenance of allele-specific methylation status of these DMRs. This is also the case for the unmethylated promoter of *Zim1*, showing no methylation in both KO and WT samples.

Second, we further tested whether the inversion has any change in the imprinting status of the individual genes within the *Peg3* domain. For this series of imprinting tests, we derived two sets of F1 hybrids through the reciprocal crossing of the male and female heterozygotes of 129/B6-mixed background with the female and male wild-types of PWD/PhJ background (**Fig 4C**). Total RNA was first isolated from the head portion of one-day-old F1 neonates, which were subsequently used for generating a set of cDNA. This set of the prepared cDNA were used for amplifying each imprinted gene of the *Peg3* domain, and the amplified PCR products were digested with several restriction enzymes that can differentiate two paternal alleles. According to the results, the paternal transmission of the inversion did not cause any change in the mono-allelic expression patterns of the individual genes within the *Peg3* domain, showing paternal expression of *Peg3*, *Usp29* and *Zfp264* whereas maternal expression of *Zim1* and *Zim2* in both KO and WT samples (3^rd^ and 4^th^ lanes in **Fig 4C**). In the case of the maternal transmission, the three genes showed no changes between KO and WT samples. However, *Zfp264* becomes bi-allelic in the KO sample (5^th^ and 6^th^ lanes in **Fig 4C**). On the other hand, *Zim2* was bi-allelic in the WT sample whereas maternally expressed in the KO sample. It is currently unknown the functional significance of these changes since these two genes have been previously shown to be somewhat sensitive to any changes in the Peg3-DMR [14, 20]. Overall, this series of analyses concluded that the inversion of the Peg3-DMR did not cause any major impact on the DNA methylation and imprinting status of the *Peg3* domain except that *Zfp264* became bi-allelic by the maternal transmission of the inversion.

### Effects on the survival and growth rates of the animals

**Fig 5:**
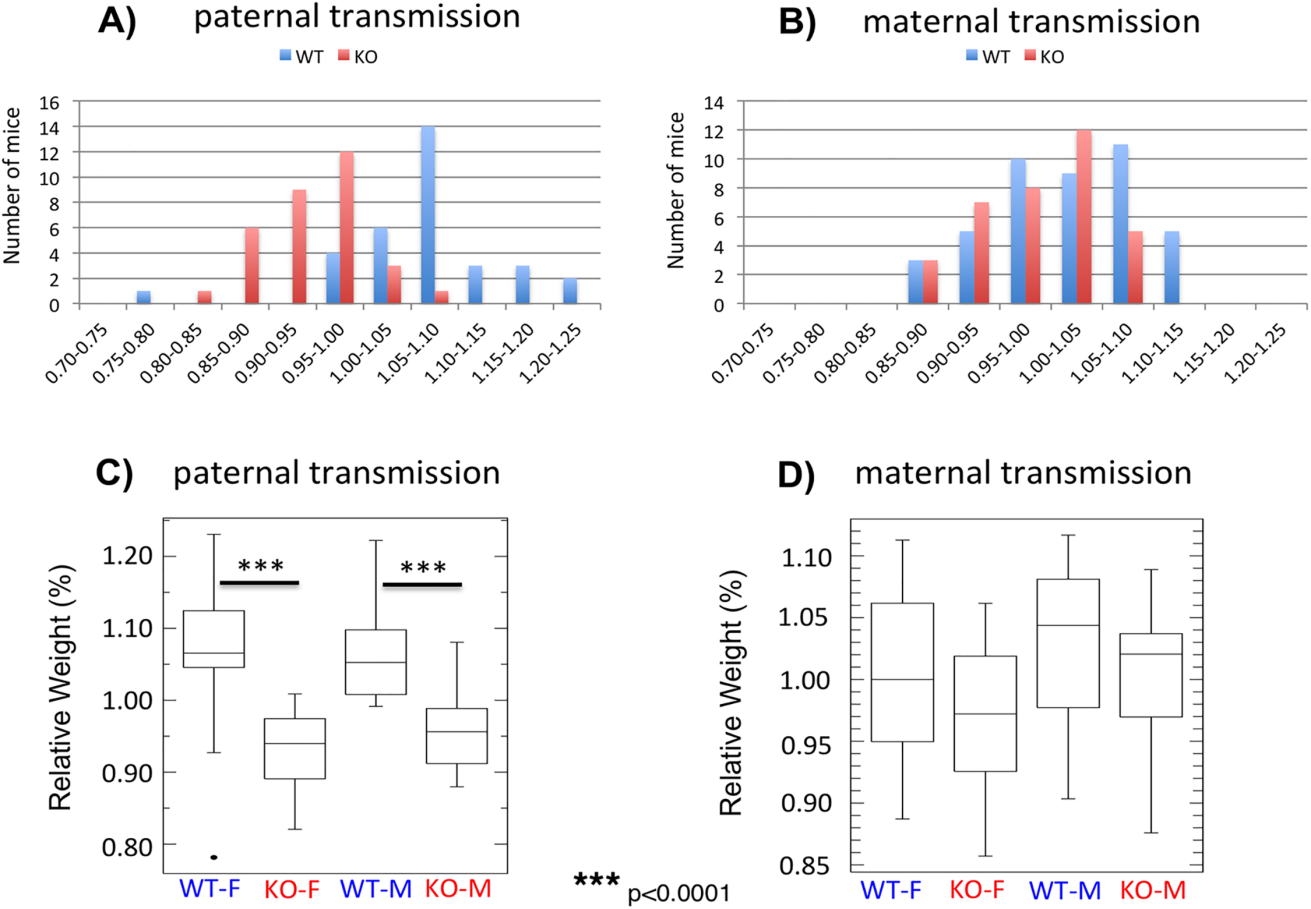
Mutational effects on the growth rates of the animals. Male and female heterozygotes for the inverted allele of the Peg3-DMR were individually bred with their wild-type littermates, deriving the pups with the paternal and maternal transmission of the inverted allele (**A**,**B**). The body weight of each pup was first divided by the average weight of a given litter, providing a percentile score for each pup. The values on the X axis indicate these percentiles, while the values on the Y axis indicate the number of mice. These weight profiles of the animals with each genotype and sex were also summarized with boxplots (**C**,**D**).

Mutational effects of the inversion of the Peg3-DMR were also analyzed at the organismal level through a series of mouse breeding experiments. We performed a set of reciprocal crossing between male and female heterozygotes with female and male wild-type littermates, deriving the pups with the paternal and maternal transmission of the inverted allele, respectively (**Fig 5A and 5B**). The health status of each pup was determined through measuring its weight, which was later converted into a percentile value through dividing its weight with the average weight of a given litter. The results derived from these breeding experiments are summarized as follows. First, the average litter sizes for the paternal and maternal transmission were 6.5 (65 pups per 10 litters) and 7.8 (78 pups per 10 litters, respectively, showing no major difference between the transmissions. The ratios of WT to KO alleles were 33 to 32 for the paternal transmission and 43 to 35 for the maternal transmission, which is very close to the mendelian ratio. Thus, the transmission of the inverted allele in either direction may have caused no lethality during development. The ratios of males to females were also 33 to 32 in the paternal transmission and 41 to 37 in the maternal transmission, indicating no bias toward one sex. Second, the average weights of one-day-old pups appeared to be affected significantly by the paternal transmission of the inverted allele. The average weights and S.D. (Standard Deviation) of the female sets were 93.2 ± 5.3% for KO and 106.3 ± 6.7% for WT, whereas those of the male sets were 95.7 ± 5.0% for KO and 105.9 ± 6.6% for WT (**Fig 5A and 5C**). This indicated that the inversion caused about 10% reduction in the weight of one-day-old pups in both sexes (Student’s t-test, *p* < 0.0001). In the case of the maternal transmission, the average weights of both females and males were very similar between KO and WT (97.2 versus 100.2% for females and 100.1 versus 103.0% for males), suggesting no major impact on the growth rates of the animals (Student’s t-test, *p* = 0.15 for females and *p* = 0.19 for males) (**Fig 5B and 5D**). Overall, this series of breeding experiments concluded that the inversion of the Peg3-DMR caused reduced growth rates when inherited as a paternal allele, but without any major impact on the survival of the animals during development.

## Discussion

**Fig 6:**
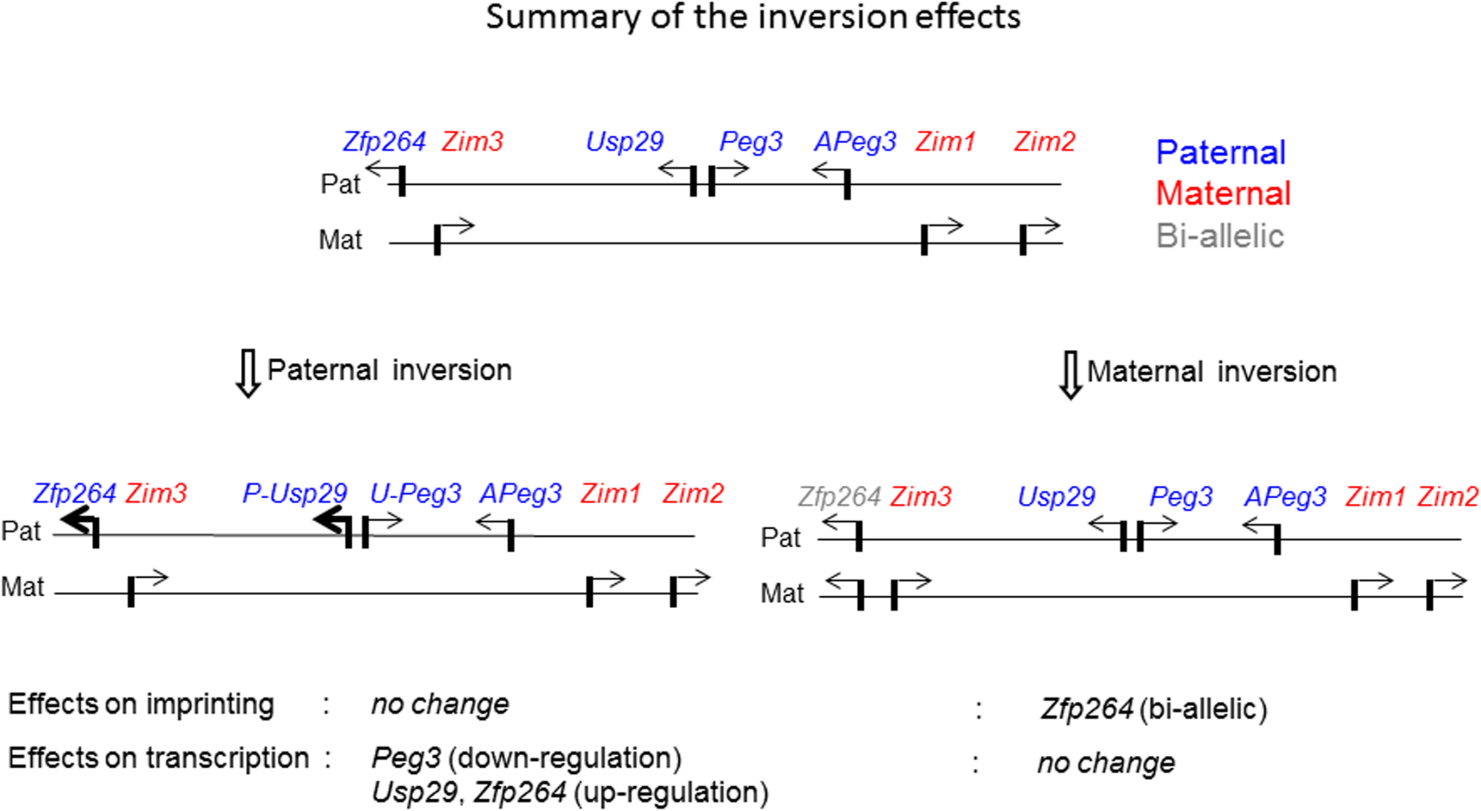
Summary of the inversion effects on the imprinting and expression of the *Peg3* domain. A schematic representation of the *Peg3* domain is shown on the upper panel. Each imprinted gene is indicated with an arrow. The paternally and maternally expressed genes are indicated with blue and red, respectively. The bi-allelically expressed genes are indicated with gray. The mutational effects by the inversion of the Peg3-DMR with the paternal and maternal transmission are summarized with two schematic diagrams on the bottom panels. The mutational effects on the imprinting status and also expression levels were summarized using the results derived from the total RNA that had been isolated from the neonatal heads with both sexes.

In the current study, the orientation of the Peg3-DMR was inverted to test its impact on the regulatory roles as an ICR for the transcription and imprinting of the *Peg3* domain. According to the results, the inversion resulted in the formation of two fusion transcripts, *U-Peg3* and *P-Usp29*, and the expression levels of these two fusion transcripts became quite different from those of the original transcripts of *Peg3* and *Usp29*. Similar to this, the expression levels of the neighboring genes were also affected (**Fig 6**). On the other hand, the DNA methylation and imprinting status of the *Peg3* domain were not affected by the inversion of this ICR. Overall, these results suggest that the orientation of the Peg3-DMR may play no roles in the DNA methylation and imprinting status, but critical for controlling the transcriptional levels of the *Peg3* domain.

The results demonstrated that the inversion of the Peg3-DMR resulted in the formation of the two fusion transcripts, *U-Peg3* and *P-Usp29* (**Fig 2**). This inversion also caused global impact on the transcriptional levels of several genes, including down-regulation of *Peg3* and up-regulation of *Usp29* and *Zfp264* (**Fig 3**). These observations are worthwhile to be discussed. First, the changes observed in the expression levels of *Zfp264* is quite interesting since this gene is localized 250-kb away from the inverted bidirectional promoter of *Peg3*/*Usp29* (**Fig 1**). This again supports the idea that the imprinted genes within the *Peg3* domain are co-regulated through an ICR, the Peg3-DMR. Also, the maternal transmission of the inversion caused bi-allelic expression of *Zfp264* although this gene has been known to be sensitive to any changes in the Peg3-DMR [20]. Second, the down and up-regulation of *Peg3* and *Usp29* by the inverted promoter were expected since these two genes are known to be expressed at different levels with the expression levels of *Peg3* being greater than those of *Usp29* [20, 21]. However, the degrees of down and up-regulation observed from the fusion transcripts were quite surprising: the inversion resulted in the 10-fold down-regulation in the *Peg3* direction and 2-fold, but not 10-fold, up-regulation in the *Usp29* direction (**Fig 3**). This unmatched, down and up-regulation between the two directions might be reflecting the difference in the genomic lengths of the transcribed regions between the two genes: a 30-kb region of *Peg3* versus a 250-kb region of *Usp29*. It is reasonable to predict that the stronger promoter of *Peg3* in the *Usp29* direction might have attracted and quenched the majority of available resource for transcription from the weaker promoter of *Usp29* in the *Peg3* direction. Thus, the transcriptional levels of *U-Peg3* became 10-fold down-regulated as compared to those of *Peg3*. On the other hand, due to the much longer size of the transcript in the *Usp29* direction, the stronger promoter of *Peg3* might have resulted in producing only 2-fold greater levels of the transcript than the original promoter of *Usp29*. Although this interpretation needs to be further tested, the inverted allele provides several insights regarding the promoter strengths and associated properties of the Peg3-DMR.

The inversion of the Peg3-DMR, surprisingly, did not cause any change in the DNA methylation and imprinting status of the *Peg3* domain except *Zfp264* (**Fig 4**). This conclusion, however, needs to be taken with some caution since the current study mainly used one tissue, neonatal heads. Also, we cannot rule out small DNA methylation changes by the inversion since the COBRA method is not sensitive enough to detect very low levels of DNA methylation changes. Nevertheless, this set of observations can be interpreted in the following manner. First, the orientation and strength of the bidirectional promoter Peg3-DMR may have no major role in establishing and maintenance of its allele-specific DNA methylation status. This is particularly interesting given the results from recent studies, demonstrating that transcription itself may be involved in establishing *de novo* DNA methylation on ICRs during gametogenesis [7, 8]. According to the results, the transcription driven by alternative promoters that are located upstream of ICRs are believed to set up several histone modification, such as H3K4me0 and H3K36me3, which in turn recruit *de novo* methyl transferase DNMT3A to the downstream ICRs [7, 8]. In the case of the *Peg3* domain, it is still unknown which alternative promoters might be involved in establishing the oocyte-specific DNA methylation on the Peg3-DMR [25]. Nevertheless, given the results from the current study, it is unlikely that the inversion of the Peg3-DMR may interfere this unknown transcription within the *Peg3* domain during oogenesis. Second, the dramatic changes in the transcriptional levels of *Peg3* and *Usp29* did not cause any changes in the DNA methylation and imprinting status of the neighboring genes, including *Zim1*, *Zim2* and *Zfp264*. This might be an indication that the transcriptional levels of *Peg3* and *Usp29* might not be a critical factor influencing the allele-specific expression patterns of the neighboring genes. This is quite surprising, particularly, for the imprinting and expression levels of *Zim1* since the promoter of this gene does not carry any allele-specific DNA methylation patterns [13]. As such, the paternal repression and subsequent maternal expression of *Zim1* has been believed to occur mainly as an outcome of the dominant paternal expression of its neighboring genes, *Peg3* and *Usp29*, through the Peg3-DMR [12]. Yet, the inversion of this dominant bidirectional promoter did not cause any change in the imprinting status of *Zim1*, thus suggesting that the imprinting status of *Zim1* is at least independent of the orientation of the Peg3-DMR. In sum, the transcription driven by the bidirectional promoter Peg3-DMR appears to play a very minimal role in determining the allele-specific expression of the *Peg3* domain.

## Materials and methods

### Ethics statement

All the experiments related to mice were performed in accordance with National Institutes of Health guidelines for care and use of animals, and also approved by the Louisiana State University Institutional Animal Care and Use Committee (IACUC), protocol #16–060.

### Generation of an inverted allele of the Peg3-DMR

Detailed information regarding the targeting vector and targeted allele with the two loxP sites flanking the 4-kb Peg3-DMR was described previously [15, 20]. The mouse strain of the 129/B6-mixed genetic background that contains two loxP sites with opposite orientation was bred with the Zp3-cre line (Jackson lab, Stock No. 003651), subsequently generating the inverted allele of the 4-kb Peg3-DMR. This mutant strain with the inverted allele was further bred with the Rosa26-FLP line (Jackson Lab, Stock No. 009086, B6.129S4-*Gt* (ROSA)*26Sor*^*tm1(FLP1)Dym*^/RainJ) to remove the *NeoR* cassette. The subsequent mutant strain with the inverted allele was used for breeding experiment in the current study.

### Mouse breeding

The male and female heterozygotes carrying the inverted allele were bred individually with female and male wild-type littermates. One-day-old pups derived from these breeding experiments were analyzed in terms of sex, genotype and body weight. Statistical significance of potential difference of litter size and average weight between breeding experiments was measured through the Student’s t-test. The sex and genotype were determined through PCR using the following two primer sets: mSry-F (5’-GTCCCGTGGTGAGAGGCACAAG-3’) and mSry-R (5’-GCAGCTCTACTCCAGTCTTGCC-3’) for the sex and Primer A (5’-TGACAAGTGGGCTTGCTGCAG-3’), B (5’-GGATGTAAGATGGAGGCACTGT-3’), C (5’-ACAACCCGGAGTTTTAGCAGAC-3’), and D (5’-AGGGGAGAACAGACTACAGA-3’) for the genotype. The genomic DNA was isolated from tail snips through incubating the tissue samples at 55^°^C in the following lysis buffer overnight (0.1 M Tris-Cl, pH 8.8, 5 mM EDTA, pH 8.0, 0.2% SDS, 0.2 M NaCl, 20 μg/ml Proteinase K). All the mice were housed at the DLAM (Division of Lab Animal Medicine) of LSU on a regular 12–12 dark-light cycle under a constant temperature 70^°^F and 50% humidity. All animals were given ad libitum access to water and Rodent Diet 5001. The nursing females were with Mouse Diet 5015. The mice were euthanized by CO2 asphixation in accordance with the rules and regulations set forth by the IACUC.

### Expression analyses and imprinting test

Total RNA was isolated from the tissues of one-day-old heads using a commercial kit (Trizol, Invitrogen). The total RNA was then reverse-transcribed using the M-MuLV kit (Invitrogen), and the subsequent cDNA was used as a template for quantitative real-time PCR. This analysis was performed with the iQ SYBR green supermix (Bio-Rad) using the *ViiA*™ *7 Real-Time PCR* System (*Life Technologies*). All qRT-PCR reactions were carried out for 40 cycles under standard PCR conditions. The analyses of the results derived from qRT-PCR were described previously [26]. Statistical significance of potential difference of expression levels of a given gene between two samples was measured through the Student’s t-test. The information regarding individual primer sequences and PCR conditions is available (**S4 File**). For imprinting test, the heterozygotes of the 129/B6 background were reciprocally crossed with the PWD/PhJ strain (Jackson Lab, Stock No. 004660). The F1 hybrid of this crossing was used for isolating total RNA. The polymorphisms and restriction enzymes used for each gene’s imprinting test are also available through the previous study [14].

### DNA methylation analysis

For DNA methylation analyses, genomic DNA from neonatal heads was treated with the bisulfite conversion protocol [23]. The isolated DNA was treated with the bisulfite conversion reaction according to the manufacturer’s protocol (EZ DNA methylation kit, Zymo Research). The converted DNA was used as a template for the PCR reaction using specific primers that were designed for amplifying each target region. The amplified products were analyzed first with COBRA (COmbined Bisulfite and Restriction Analysis) [24]. The information regarding the sequences of oligonucleotides and the PCR conditions for each genomic region is also available (**S4 File**).

## Supporting information

S1 File This file contains the results from a series of RT-PCR analyses detecting the two fusion transcripts using the two sets of cDNA that had been prepared from the neonatal pups with the paternal and maternal transmission of the inverted allele. (TIF)

S2 File This file contains the results from a series of RT-PCR analyses detecting potential fusion transcripts between the bidirectional promoter and the adjacent genes, *Zim1, Zim2* and *Zfp264*. (TIF)

S3 File This file contains the results from a series of qRT-PCR analyzing the potential effects of the maternal transmission of the inverted allele on the expression levels of the imprinted genes within the *Peg3* domain. (TIF)

S4 File This file contains a list of PCR primers used for the current study. This includes the primer sets for RT-PCR and COBRA analyses.
(XLSX)
