# Supplementary figures and images for: Inversion of the imprinting control region of the Peg3 domain
Source: PLoS One. 2017 Jul 18;12(7):e0181591. doi: 10.1371/journal.pone.0181591 (PMC5515438; doi:10.1371/journal.pone.0181591)

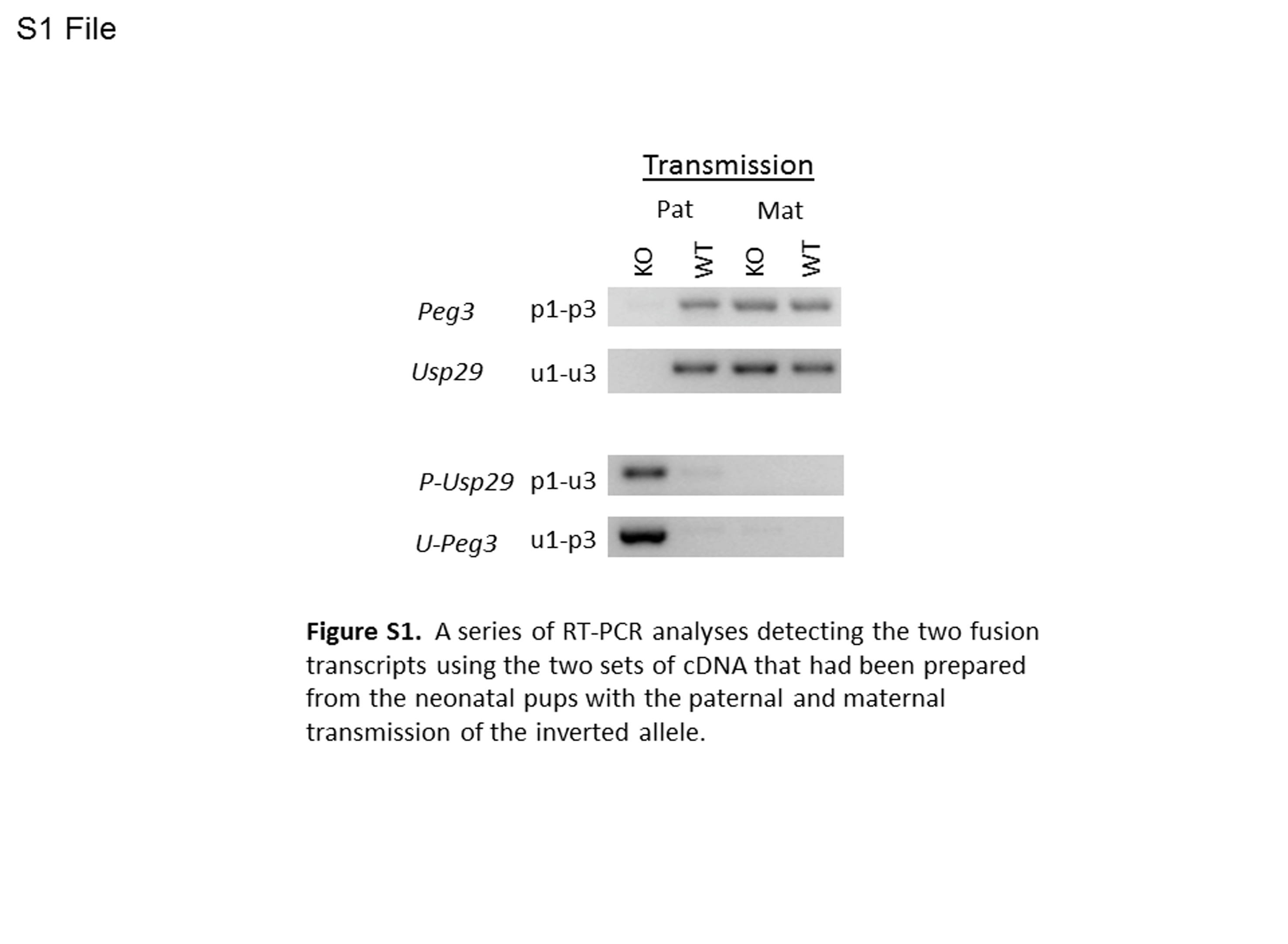

Supplement: S1 File — (TIF) [file pone.0181591.s001.TIF]

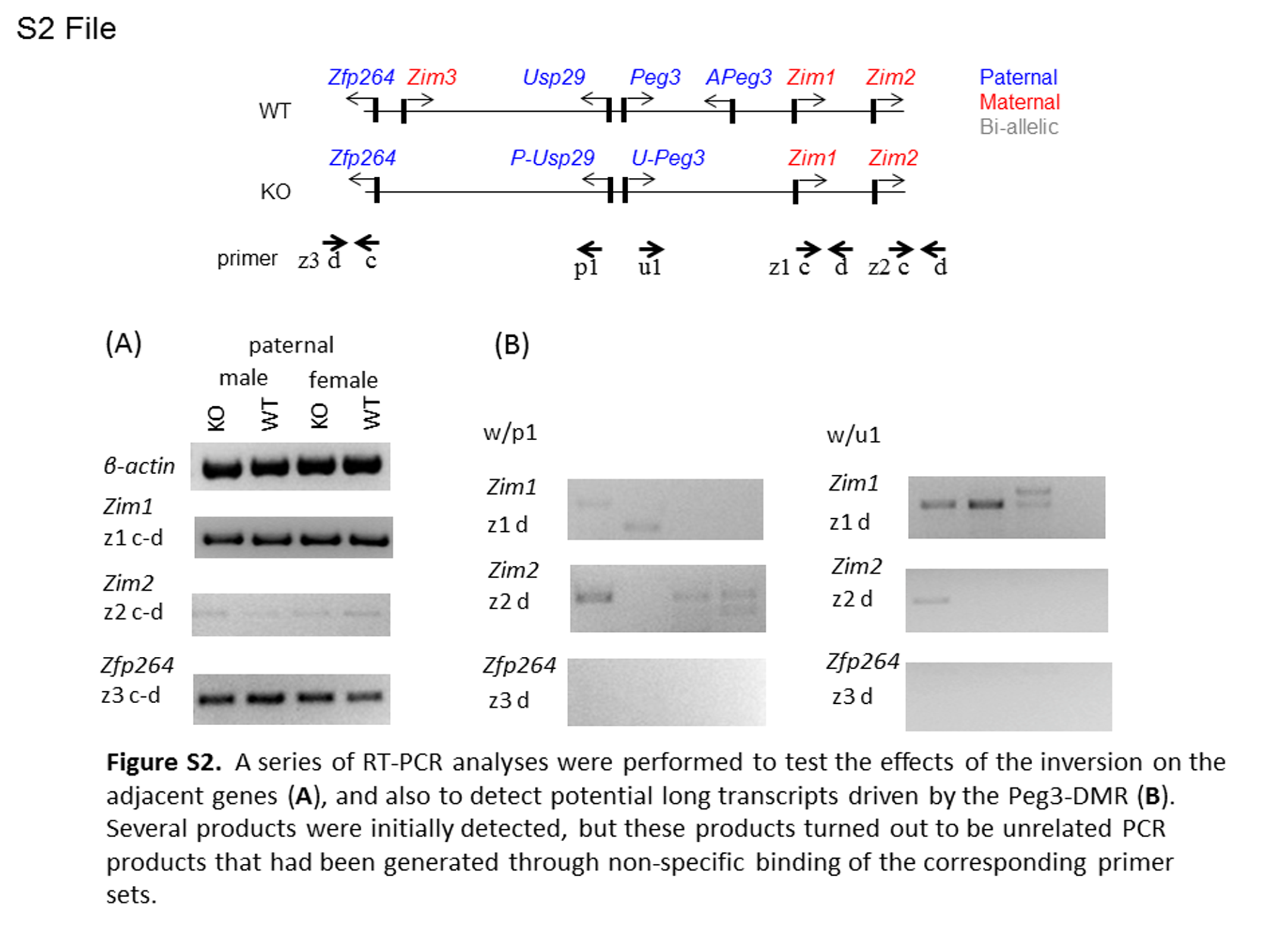

Supplement: S2 File — (TIF) [file pone.0181591.s002.TIF]

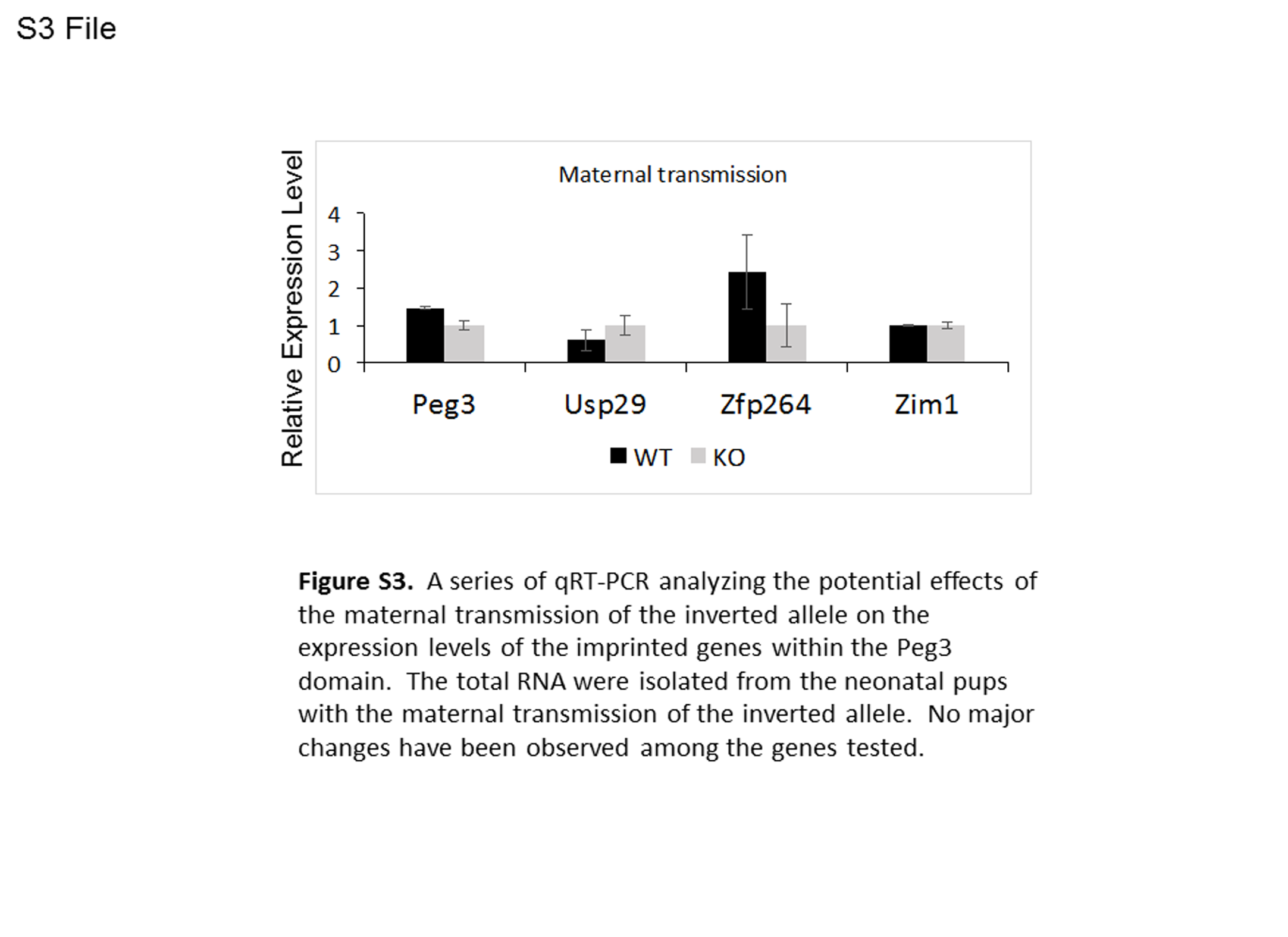

Supplement: S3 File — (TIF) [file pone.0181591.s003.TIF]
